# Characterization and Therapeutic Potential of Induced Pluripotent Stem Cell-Derived Cardiovascular Progenitor Cells

**DOI:** 10.1371/journal.pone.0045603

**Published:** 2012-10-09

**Authors:** Ali Nsair, Katja Schenke-Layland, Ben Van Handel, Denis Evseenko, Michael Kahn, Peng Zhao, Joseph Mendelis, Sanaz Heydarkhan, Obina Awaji, Miriam Vottler, Susanne Geist, Jennifer Chyu, Nuria Gago-Lopez, Gay M. Crooks, Kathrin Plath, Josh Goldhaber, Hanna K. A. Mikkola, W. Robb MacLellan

**Affiliations:** 1 Department of Medicine and Physiology, Cardiovascular Research Laboratory, David Geffen School of Medicine, University of California Los Angeles, Los Angeles, California, United States of America; 2 Department of Cell and Tissue Engineering, Fraunhofer Institute for Interfacial Engineering and Biotechnology IGB, Stuttgart, Germany; 3 Department of Thoracic and Cardiovascular Surgery, Eberhard Karls University, Tübingen, Germany; 4 Department of Pathology and Laboratory Medicine, David Geffen School of Medicine, University of California Los Angeles, Los Angeles, California, United States of America; 5 Department of Molecular, Cell and Developmental Biology, David Geffen School of Medicine, University of California Los Angeles, Los Angeles, California, United States of America; 6 The Edythe and Eli Broad Center for Regenerative Medicine and Stem Cell Research, David Geffen School of Medicine, University of California Los Angeles, Los Angeles, California, United States of America; 7 Cedars-Sinai Heart Institute, Los Angeles, California, United States of America; 8 Department of Biochemistry and Molecular Biology and Institute for Stem Cell and Regenerative Medicine, University of Southern California, Los Angeles, California, United States of America; 9 Institute for Stem Cell and Regenerative Medicine, University of Washington, Seattle, Washington, United States of America; Centro Cardiologico Monzino, Italy

## Abstract

**Background:**

Cardiovascular progenitor cells (CPCs) have been identified within the developing mouse heart and differentiating pluripotent stem cells by intracellular transcription factors Nkx2.5 and Islet 1 (Isl1). Study of endogenous and induced pluripotent stem cell (iPSC)-derived CPCs has been limited due to the lack of specific cell surface markers to isolate them and conditions for their *in vitro* expansion that maintain their multipotency.

**Methodology/Principal Findings:**

We sought to identify specific cell surface markers that label endogenous embryonic CPCs and validated these markers in iPSC-derived Isl1^+^/Nkx2.5^+^ CPCs. We developed conditions that allow propagation and characterization of endogenous and iPSC-derived Isl1^+^/Nkx2.5^+^ CPCs and protocols for their clonal expansion *in vitro* and transplantation *in vivo*. Transcriptome analysis of CPCs from differentiating mouse embryonic stem cells identified a panel of surface markers. Comparison of these markers as well as previously described surface markers revealed the combination of Flt1^+^/Flt4^+^ best identified and facilitated enrichment for Isl1^+^/Nkx2.5^+^ CPCs from embryonic hearts and differentiating iPSCs. Endogenous mouse and iPSC-derived Flt1^+^/Flt4^+^ CPCs differentiated into all three cardiovascular lineages *in vitro*. Flt1^+^/Flt4^+^ CPCs transplanted into left ventricles demonstrated robust engraftment and differentiation into mature cardiomyocytes (CMs).

**Conclusion/Significance:**

The cell surface marker combination of Flt1 and Flt4 specifically identify and enrich for an endogenous and iPSC-derived Isl1^+^/Nkx2.5^+^ CPC with trilineage cardiovascular potential *in vitro* and robust ability for engraftment and differentiation into morphologically and electrophysiologically mature adult CMs *in vivo* post transplantation into adult hearts.

## Introduction

Despite therapeutic advancements, cardiovascular disease remains a major cause of morbidity and mortality worldwide. Although current therapies slow the progression of cardiovascular disease, there are few if any options to reverse or repair damaged myocardium. Unfortunately, adult cardiac myocytes (CMs) lack the ability to divide and replace those that are damaged after injury in any clinically significant manner [1]. Investigators have been exploring the feasibility of directly injecting stem cells into the heart for therapeutic cell transplantation and regeneration. While multiple animal studies have demonstrated the ability of adult stem cells to improve left ventricular function, long-lasting effects, CM differentiation or even engraftment of injected cells has been more difficult to establish [2], [3]. Likewise, early human clinical trials testing the efficacy of adult stem cell therapy to restore perfusion and mechanical function to the heart after myocardial infarction (MI), although promising, have had variable results [4]. Since most preclinical studies have demonstrated very low rates of cardiac differentiation when using these cells [5], there is increasing consensus that transplanted adult stem cells may have a limited capacity for true cardiac regeneration and their beneficial effects are more likely related to paracrine mechanisms [6]. This highlights the need for cell types that can provide long-lasting engraftment and myogenesis either alone or in combination with existing cell types. Embryonic stem cells (ESCs) are a reliable source of authentic CMs, but issues of immunogenicity, oncogenic risk and ethical concerns have hampered their clinical translation. Recent advances in stem cell biology to induce pluripotency in somatic cells make the potential of autologous, regenerative strategies a viable possibility [7]. However, translating the promise of iPSCs into a viable therapy will require the identification and characterization of appropriate iPSC-derived progenitor cells. We believe that the optimal cell type would be lineage-committed, multipotent CPCs that satisfy the need for multilineage differentiation while limiting the oncogenic risk of injecting undifferentiated iPSCs or ESCs.

Recently, a multipotent CPC was identified based on the expression of transcription factors Isl1^+^ and Nkx2.5^+^ [8], [9] in ESCs and fetal hearts; however, surface markers to identify and enrich for these Isl1^+^/Nkx2.5^+^ CPCs are neither specific nor uniformly agreed upon. Previously described cell surface proteins Flk1 and Kit oncogene (c-kit), which have been used in combination to identify mouse CPCs, are not specific markers for endogenous CPCs [10] since Flk1 is broadly expressed developmentally on all cardiovascular cell types and not limited to Isl1^+^/Nkx2.5^+^ CPCs [11]. Genetically modifying CPCs with integrating viruses to express fluorescent markers under the control of Isl1 or Nkx2.5 promoters has also been used to identify these CPCs [12]. However, this would complicate their use clinically in human trials due to potential oncogenic risk incurred by genomic manipulation. Therefore, the ability to utilize CPCs derived from human iPSCs therapeutically will require the identification of surface markers to isolate and enrich for Isl1^+^/Nkx2.5^+^ CPCs without genetic manipulation [10]. Furthermore, it has proven difficult to propagate and expand progenitor cells while simultaneously maintaining their multipotent differentiation potential, hampering attempts to generate sufficient numbers of CPCs *ex vivo* to study and/or use in regenerative therapies. Thus, the lack of specific cell surface markers that identify Isl1^+^/Nkx2.5^+^ CPCs in an unmodified form and the lack of appropriate conditions to expand them *in vitro* remains one of the major roadblocks facing translational clinical applications of CPCs [10].

In this study, we attempted to identify cell surface markers that are specific to and allow enrichment of Isl1^+^/Nkx2.5^+^ CPCs. We identified Flt1 and Flt4 as a novel cell surface marker combination that is specific to and enriches for mouse endogenous and iPSC-derived CPCs. These Flt1^+^/Flt4^+^ CPCs have trilineage cardiovascular potential and can be expanded *ex vivo*, feeder-free, using small molecule inhibitors. These Flt1^+^/Flt4^+^ CPCs not only have the ability to be clonally expanded and differentiate into all three cardiovascular lineages *in vitro*, their *in vivo* differentiation potential post-transplantation appears to be preferentially towards authentic adult CMs both morphologically and electrophysiologically. Thus, utilizing the approaches outlined in this report, the combination of surface markers Flt1 and Flt4 enrich for iPSC-derived CPCs, which could be a unique source of multipotent progenitor cells for cardiac cell therapy.

## Materials and Methods

All surgeries were performed under the supervision and with approval of the University of California, Los Angeles Animal Review Committee.

### Microarray Analysis for Identification of CPC Surface Markers

RNA samples from mouse ESCs or ESC-derived Flk1^+^ cells were analyzed using Illumina Microarrays and have been previously reported [13]. Briefly, biotinylated cRNA was prepared using the Illumina RNA Amplification Kit (Ambion). Samples were used for hybridization on a Sentrix MouseRef-8 Expression BeadChip System (Illumina). The microarray raw data was analyzed using BeadStudio 3.2.2 (Illumina). For additional details, see the online supplementary methods (Methods S1).

### Immunohistochemistry of Endogenous CPCs

Immunofluorescent staining (antibodies listed in Table S1) of paraffin-embedded tissue sections of E15.5 mouse embryonic hearts was performed as previously described [13]. Fluorescence images were acquired using a confocal TCS SP2 AOBS laser-scanning microscope system (Leica). Images were processed with Adobe Photoshop CS3 (Adobe). Additional details are available in the online supplementary methods (Methods S1).

### Isolation and Expansion of Endogenous CPCs

Mouse hearts were dissected from E15.5 embryos (CF1-mice; Charles River). To harvest CPCs, heart tissue was minced and subjected to collagenase digestion. Murine cells were maintained on Mitomycin-C treated, primary mouse embryonic fibroblasts (MEF) in IQ1-supplemented medium [14]. Cells were expanded for 8 days and the Flt1^+^/Flt4^+^ cells isolated by indirect magnetic-activated cell sorting (MACS) (Stem Cell Technologies) or fluorescence-activated cell sorting (FACS) using conjugated Flt1 and Flt4 antibodies (Abcam). For more details, see the online supplementary methods (Methods S1).

### Isolation of iPSC-derived CPCs

Murine iPSCs were generated from C57/BL6 mice as previously described [13], transduced to constitutively express green fluorescent protein (GFP), and expanded on Mitomycin C inactivated MEFs and maintained in an undifferentiated state in LIF medium. Mouse iPSCs were differentiated on Collagen IV coated plates for 4 days and dissociated into single cell suspension with 0.05% trypsin (Invitrogen). Flt1^+^/Flt4^+^ cells were isolated by indirect MACS or FACS using conjugated Flt1 and Flt4 antibodies (Abcam). For more details, see the online supplementary methods (Methods S1).

### Differentiation Assays

For *in vitro* differentiation assays, purified Flt1^+^/Flt4^+^ cells were plated on fibronectin-coated culture slides (BD Bioscience) in either alpha-MEM (cardiac differentiation), PDGF medium (smooth muscle differentiation), or VEGF medium (endothelial differentiation) as previously described [13]. For additional details, see the online supplementary methods (Methods S1).

### Gene Expression Analysis

Total RNA was extracted from mouse hearts, mouse ESCs, the Flt1^−/^Flt4^−^ negative cell fraction, as well as from mouse undifferentiated and differentiated Flt1^+^/Flt4^+^ cells. Semi-quantitative PCR was performed as previously described [13]. All mouse primer sequences have been previously published [13]. Primer sets specific for mouse Flt1 and Flt4 were purchased from R&D Systems. For additional details, see the online supplementary methods (Methods S1).

### Clonal Expansion and Differentiation of CPCs

Skin fibroblasts from C57/BL6 mice were reprogrammed as described [13] and stably transfected with a constitutively expressed GFP. Single cell FACS sorting was performed for the Flt1^+^/Flt4^+^ population on the ARIA-II Flow Cytometer. After cell recovery overnight, Flt1^+^/Flt4^+^ cells were propagated feeder-free in ESGRO media (Millipore), which was supplemented with IQ-1 (Calbiochem) and ROCK inhibitor Thiazovivin (Stemgent) on fibronectin-coated plates (BD Bioscience). After four weeks, colonies arising from single cell clones were dissociated and replated onto fibronectin-coated culture slides (BD Bioscience) in differentiation media for fourteen days as described before [13]. For additional details, see the online supplementary methods (Methods S1).

### *In vivo* Transplantation of Cardiac Progenitor Cells

GFP^+^ mouse iPSCs were differentiated and GFP^+^ Flt1^+^/Flt4^+^ CPCs were isolated by FACS sorting as described above. Post-sort, Flt1^+^/Flt4^+^ CPCs were recovered overnight at 4°C in CPC pro-survival media [15] prepared with ESGRO media (Millipore) and pro-survival factors with growth factor–reduced Matrigel (BD bioscience), supplemented with 100 µM ZVAD (Calbiochem), 50 nM Bcl-X_L_ BH4 (Calbiochem), Cyclosporine A 200 nM (Wako Pure Chemicals), 100 ng/ml IGF-1 (Peprotech), and 50 µM pinacidil (Sigma). Flt1^+^/Flt4^+^ CPCs were recovered overnight in CPC pro-survival at 4°C. Cells were then suspended at a concentration of 50,000 cells per 50 µl of CPC pro-survival media and injected into the anterior wall of the left ventricle at 10 µl per injection site for a total of 5 injections. Sham injections with pro-survival media were performed on control mice. 28 days post injection, the animals were sacrificed, hearts were harvested, fixed according to standard protocols and immunofluorescence imaging was performed. For additional details, see the online supplementary methods (Methods S1).

### Isolation and Electrophysiological Assessment of GFP^+^ Flt1^+^/Flt4^+^-derived CMs Post *in vivo* Transplant of CPCs

Adult mice were injected with 800 µl of heparin (5,000 units/ml) intraperitonealy twenty minutes prior to harvesting of hearts. Animals were anesthetized with isoflurane and hearts excised via a thoracotomy. Single ventricular myocytes were enzymatically separated using collagenase (3 mg/ml, Type II collagenase; Gibco BRL) and protease (0.3 mg/ml, Type XIV protease; Sigma-Aldrich). Following isolation, dissociated cells were washed three times and resuspended at room temperature in modified Tyrode’s solution, containing (in mmol/L): 136 NaCl, 5.4 KCl, 10 HEPES, 1.0 MgCl_2_, 0.33 NaH2PO4, 0.5 CaCl_2_, 10 glucose (pH 7.4). Isolated CMs were loaded with 2 mM rhod-2AM and 1 µl Pluronic F-127 (both from Molecular Probes). Cells were then placed in an experimental chamber (0.5 ml) filled with normal Tyrode’s solution. A Leica SP5 resonant LSCM system (Leica) was used for confocal imaging. Cells were whole cell patch-clamped and action potentials were induced at a rate of 0.2 Hz using the current clamp mode of an Axopatch900A amplifier (Molecular Devices, Sunnyvale, CA). Calcium transients were simultaneously recorded. For additional details, see the online supplementary methods (Methods S1).

### Statistical Analysis

All data were presented as mean ± standard error of the mean (SEM). Statistical significance was assessed by Student’s *t* test or ANOVA with Tukey’s multiple comparison test. *P*-values less than 0.05 were defined as statistically significant.

## Results

### The Flt1^+^/Flt4^+^ Cell Population in Developing Hearts is Enriched for Isl1^+^/Nkx2.5^+^ Cardiac Progenitors

**Figure 1:**
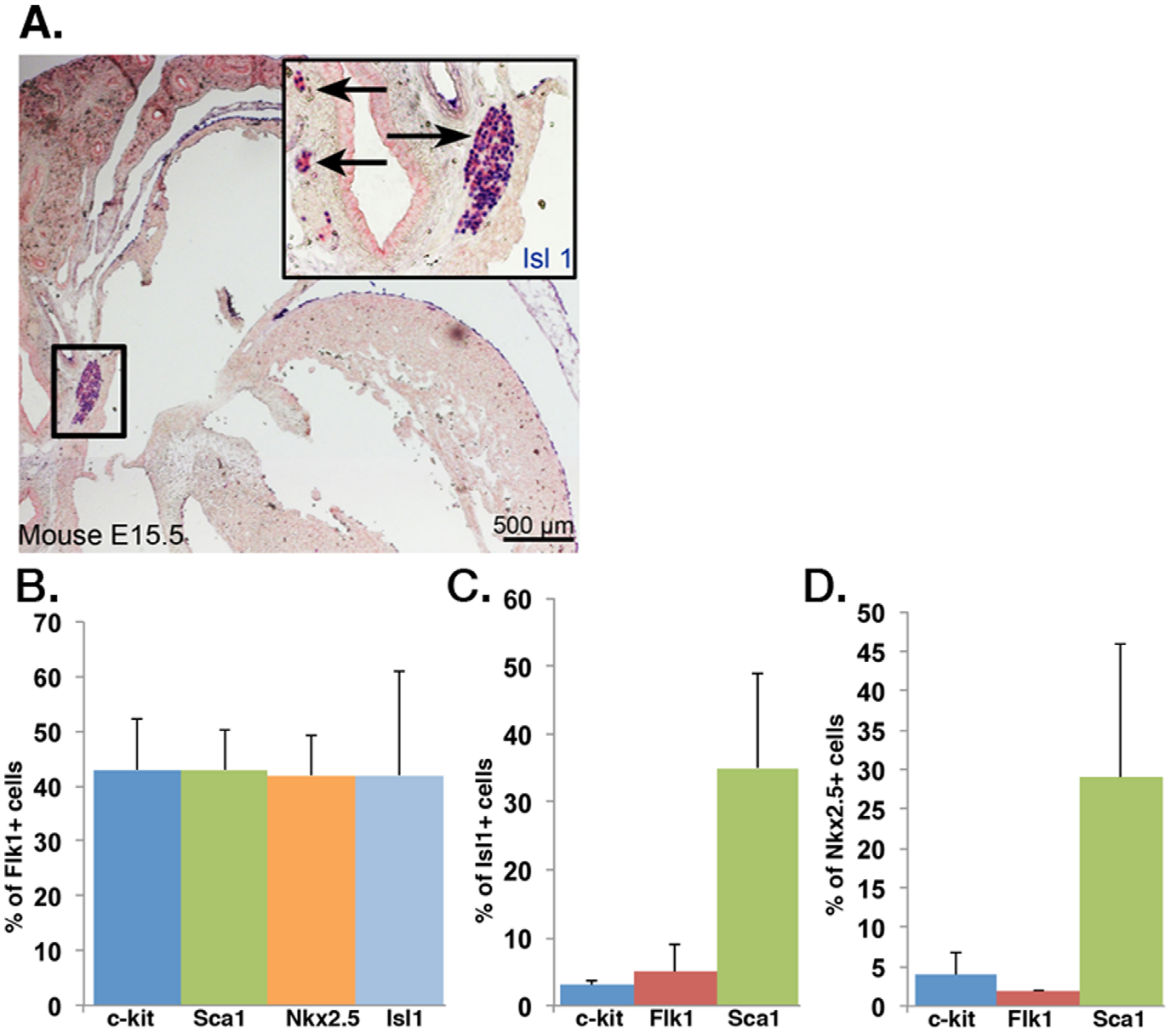
Flk1 is not a specific marker for endogenous and mouse ESC-derived Isl1^+^ CPCs. (A) Immunohistochemical staining of E15.5 mouse heart identifies Isl1-expressing CPCs (blue) located in niches in outflow tracts. (B) FACS analysis of mouse ESC-derived Flk1^+^ cells reveals a heterogeneous Flk1^+^ population with low enrichment for Isl1 (light blue bar) and Nkx2.5 (orange bar) cells (n = 3). (C & D) FACS analysis of differentiated mouse ESCs reveals that Flk1 represents <10% of Isl1^+^ cells (C; n = 3) and <5% of Nkx2.5^+^ cells (D; n = 3).

Cardiovascular progenitors that contribute to all three cardiovascular lineages identified by the transcription factor Isl1 have been described in the developing human and mouse fetal heart [16]. Although Flk1 has been widely accepted as a cell surface marker to identify CPCs, at least in differentiating pluripotent stem cells [17], it is not specific *in vivo*. Flk1 is a marker of early cardiogenesis and, consistent with genetic tracking studies [11], embryonic hearts express Flk1 diffusely making it a poor marker to selectively identify and isolate endogenous CPCs (Fig. 1A). FACS analysis demonstrated that Flk1 is also a nonspecific marker for ESC-derived Isl1^+^/Nkx2.5^+^ CPCs as it labeled a heterogeneous population of cells in differentiating mouse ESCs (Fig. 1B). Less than 10% of Isl1^+^ cells were Flk1^+^ and less than 5% of Nkx2.5^+^ cells were Flk1^+^ (Fig. 1C, 1D). c-kit (CD117), another proposed CPC marker, was equally nonspecific. Although Sca1 was able to identify the greatest percentage of Isl1 or Nkx2.5 cells, it still only captured a minority of these populations and is not expressed on human cells.

**Figure 2:**
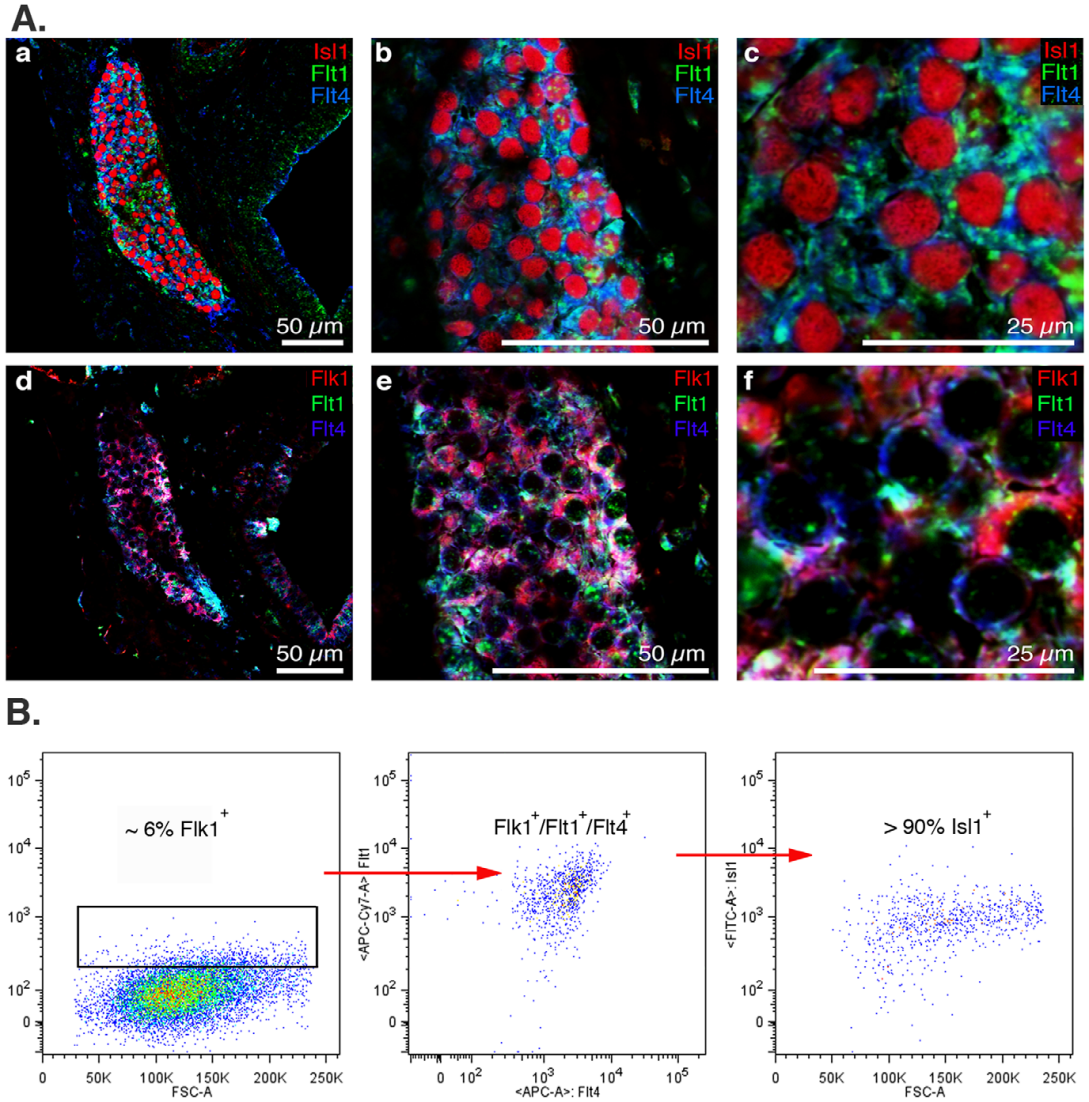
Novel surface marker combination Flt1 and Flt4 specifically label endogenous Isl1^+^ CPCs in E15.5 mouse hearts. (Aa-c) Immunofluorescence imaging of E15.5 mouse fetal hearts shows that surface markers Flt1 (green) and Flt4 (blue) co-label Isl1^+^ CPCs (red) in specialized niches. (Ad-f) Flt1^+^ (green) and Flt4^+^ (blue) double positive cells are also Flk1^+^ (red). (B) FACS analysis of digested E15.5 mouse hearts: Flk1^+^/Flt1^+^/Flt4^+^ are greater than 90% Isl1^+^ confirming Flt1 and Flt4 in combination enrich for Isl1^+^ CPCs.

To identify surface markers that could be used to specifically label and enrich for CPCs, we first examined the transcriptome of mouse ESC-derived Flk1^+^ cells, which includes a subpopulation of cardiovascular progenitor cells, which have been shown to label a trilineage CPC from differentiating embryonic stem cells [13]. Reanalysis of this microarray data identified six additional cell surface markers that have been associated with various stem or progenitor cell populations. In addition to Flk1, the two other members of the vascular endothelial growth factor receptor (VEGFR) family, VEGFR1 and VEGFR3 (Flt1 and Flt4) were identified. To compare the utility of these cell surface markers in identifying and enriching for the Isl1^+^ population, we performed a detailed FACS analysis of cells dissociated from E15.5 mouse embryonic hearts (Table 1). We examined the sensitivity and specificity of Flt1, Flt4, Flk1, c-kit, CD31 (PECAM-1 or Platelet Endothelial Cell Adhesion Molecule), CD34, and PDGFR-α singly or in combination to identify and enrich for endogenous Isl1^+^ putative CPCs. No cell surface marker was sufficient by itself to identify the Isl1^+^ population (Table 1). When different combinations of surface markers were compared, the combination of Flt1/Flt4 was the most specific for identifying and enriching for the Isl1^+^ population, as 89.4% of Flt1^+^/Flt4^+^ cells were Isl1^+^ (Table 1). Furthermore, Flt1^+^/Flt4^+^ identified 64.5% of the Isl1^high^ population, which we believe represents the true undifferentiated CPC population [16] as Isl1 expression decreases as CPCs differentiate into progeny cells. In contrast, only 7.43% of Flk1^+^ cells were Isl1^+^ and the combination of Flk1 and PDGFR-α, which has been previously described to isolate cardiac progenitors from differentiating ESCs [16], only identified 23.8% of the Isl1^high^ CPC population. As well, endogenous Isl1^+^ progenitors were negative for SSEA-1, which has been reported to enrich for primate ESC-derived Isl1^+^ cells [18]. To validate the expression of these cell surface markers on putative CPCs, we immunostained E15.5 mouse hearts for Isl1 and all three VEGF receptor family members Flk1, Flt1 and Flt4. Flt1 and Flt4 expression preferentially co-localized with Isl1^+^ cells (Fig. 2Aa–f). Although neither Flt1 nor Flt4 are exclusively expressed on Isl1^+^ cells, their co-expression reliably and specifically identified Isl1^+^ cells in the developing mouse heart allowing for identification of the endogenous Isl1^+^ population by FACS (Fig. 2B).

**Table 1:** FACS analysis of cells dissociated from E15.5 mouse hearts.

Cell surface marker/s	% surface marker/s- labeledcells that are Isl1^+^	% Isl1^high^ cells labeled bysurface marker/s	% cells in E15.5 heart labeledby surface marker/s
Flt1^+^	19.7%	69.7%	7.6%
Flt4^+^	19.4%	79.7%	15.9%
Flk1^+^	7.43%	56.9%	27.2%
c-kit^+^	<1%	N/A	0.7%
CD31^+^	5.4%	71.6%	49.8%
CD34^+^	6.8%	87.6%	42.9%
Flk1^+^/PDGR-α^+^	12.1%	23.8%	2.1%
Flt1^+^/Flt4^+^	89.4%	64.5%	5.89%
Flk1^+^/Flt4^+^	8.55%	70.6%	10.3%
Flt1^+^/CD31^+^	19.4%	63.2%	9.58%
Flt4^+^/CD31^+^	11.4%	49.8%	12.4%

FACS analysis of E15.5 mouse embryonic hearts for different cell surface marker/s identified by microarray analysis of mouse ESC-derived Flk1^+^ cells. Flt1/Flt4 combination is the most specific to identify and enrich for Isl1^+^ cells in the E15.5 heart.

We performed immunostaining of E15.5 mouse hearts for Isl1 and for the pan-neuronal marker Microtubule-associated protein-2 (Map2) and the peripheral ganglion protein Neurofilament, Heavy Polypeptide (NEFH)(Figure S1). Some Isl1^+^ cells were positive for Map2, indicating their potential neural crest lineage, but fewer were positive for NEFH. This is in line with recent reports that Isl1^+^ cells in the heart, later in gestation, represent cells from the second heart field, cardiac neural crest as well as cardiac ganglia.

### Isolated Endogenous Flt1^+^/Flt4^+^ Cells Expanded *in vitro* Display Trilineage Differentiation Potential

**Figure 3:**
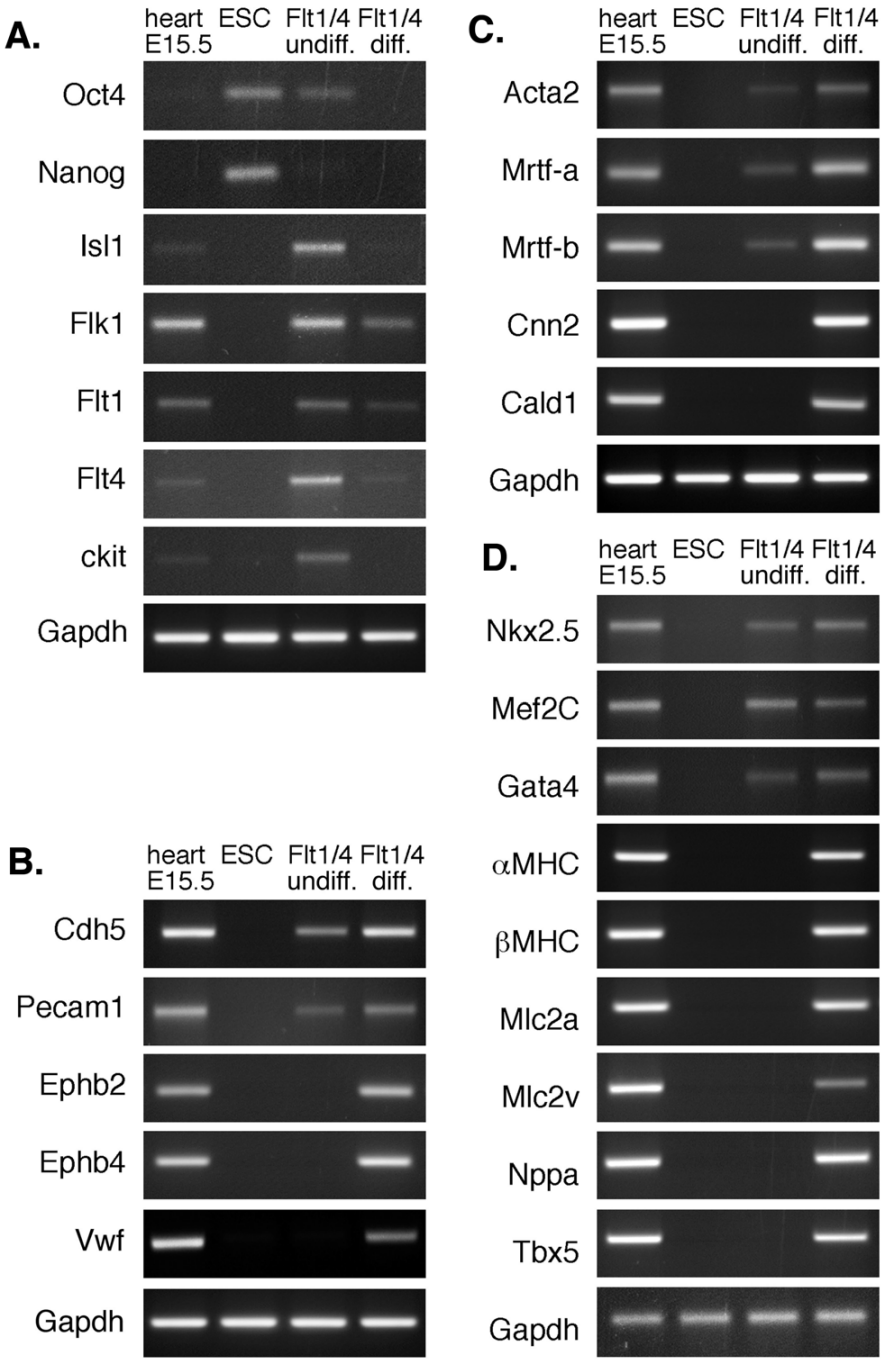
Endogenous Flt1^+^/Flt4^+^ cells are multipotent CPCs that differentiate into all three lineages of the cardiovascular system. (A) Semi-quantitative PCR analysis shows that undifferentiated murine Flt1^+^/Flt4^+^ cells express markers of mesodermal and cardiovascular progenitor cells that are down-regulated during differentiation. (B–D) Differentiating murine Flt1^+^/Flt4^+^ cells show up-regulation of genes characteristic of the (B) endothelial, (C) smooth muscle and (D) cardiac lineages.

To confirm that the Flt1^+^/Flt4^+^ population we identified were indeed CPCs, we assessed their ability to differentiate into the three cardiovascular lineages. Endogenous mouse Flt1^+^/Flt4^+^ cells were isolated and expanded *in vitro* and then exposed to specific differentiation conditions. The undifferentiated Flt1^+^/Flt4^+^ population expressed high levels of progenitor cell markers including Isl1, Nkx2.5, Flk1, Flt1, Flt4 and c-kit, but not genes associated with differentiated cardiovascular cells (Fig. 3A). Differentiated Flt1^+^/Flt4^+^ cells down-regulated these CPC markers and up-regulated genes expressed in endothelial cells (EC) and smooth muscle cells (SMC) as well as CMs (Fig. 3B, 3C and 3D). Thus, the Flt1/Flt4 cell surface marker combination identifies a progenitor population in developing mouse hearts that is able to differentiate into all three cardiovascular cell types.

To quantify the *in vitro* differentiation potential of Flt1^+^/Flt4^+^ CPCs when compared to the cell population enriched for by the marker Flk1, we isolated mouse ESC-derived Flt1^+^/Flt4^+^ and Flk1^+^ cells and compared them directly. Both cell populations were plated in equal numbers onto fibronectin-coated plates post-FACS sorting and exposed to identical CM differentiation conditions for 14 days. 73±4.8% of the cells from differentiated Flt1^+^/Flt4^+^ CPCs were Troponin C (TropC) positive compared to only 43±5.7% of the Flk1^+^ population. Furthermore, to assess the ability of Flt1 and Flt4 to capture the majority of the CPC population from differentiating ESCs, the negative fraction population of Flt1^−/^Flt4^−^ cells was collected and exposed to the same differentiation conditions. Flt1^−/^Flt4^−^ cells had no cardiogenic differentiation potential when exposed to identical differentiation conditions. The combination of Flt1/Flt4 cell surface markers functionally enriches for a progenitor cell population capable of a more robust *in vitro* CM differentiation when compared to the cell population identified by the previously described marker Flk1.

### Single Flt1^+^/Flt4^+^ Cells are Authentic, Multipotent Cardiac Progenitor Cells that can be Clonally Expanded and Differentiated

**Figure 4:**
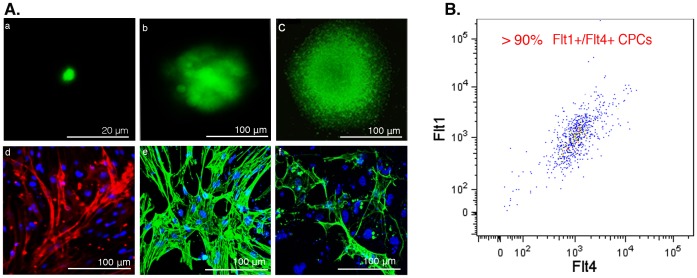
Mouse iPSC-derived GFP^+^ Flt1^+^/Flt4^+^ CPCs can be clonally expanded while maintaining phenotype and multipotency. (Aa-c) Immunofluorescence imaging of live GFP^+^ Flt1^+^/Flt4^+^ single cell CPCs during clonal expansion: (a) single cell post FACS sort, day 1, (b) same colony on day 14 and (c) on day 30. (Ad-f) CPCs demonstrate trilineage cardiovascular differentiation potential post-clonal expansion. Immunofluorescence imaging of clonally expanded Flt1^+^/Flt4^+^ CPC colonies fourteen days post-differentiation demonstrating (d) TropC-expressing CMs, (e) α-SMA expressing smooth muscle cells, and (f) CD31 expressing endothelial cells. Cell nuclei are identified with DAPI. (B) FACS analysis thirty days post-clonal expansion confirms CPCs maintain their Flt1^+^/Flt4^+^ CPC phenotype.

To determine the clonal potential of Flt1^+^/Flt4^+^ cells, we performed single cell FACS sorting of mouse iPSC-derived Flt1^+^/Flt4^+^ cells onto fibronectin-coated plates. Post-FACS, these cells were clonally expanded in the presence of IQ-1, a selective β-catenin/p300 inhibitor [14], [19] and Thiazovivn, a ROCK inhibitor [20]. After plating Flt1^+^/Flt4^+^ cells at one cell per well, 54.2% (26 of 48) cells survived and formed colonies. After four weeks, homogeneous clusters of cells (Fig. 4Aa–c), of which >90% remained Flt1^+^/Flt4^+^ (Fig. 4B) were apparent. Post expansion, colonies were dissociated and replated onto fibronectin-coated plates and exposed to differentiation conditions as described above. Spontaneously beating colonies were observed 13 days post differentiation (Movie S1). Immunostaining of six independent clones chosen randomly from colonies formed revealed that they were all capable of forming spontaneously beating colonies and differentiating into CMs (Fig. 4Ad, Figure S2), SMCs (Fig. 4Ae) and ECs (Fig. 4Af). Thus, iPSC-derived Isl1^+^/Flt1^+^/Flt4^+^ cells can be clonally expanded for a minimum of thirty days while maintaining their surface marker phenotype and trilineage differentiation potential.

### *In vivo* Transplantation and Engraftment of Flt1^+^/Flt4^+^ CPCs

**Figure 5:**
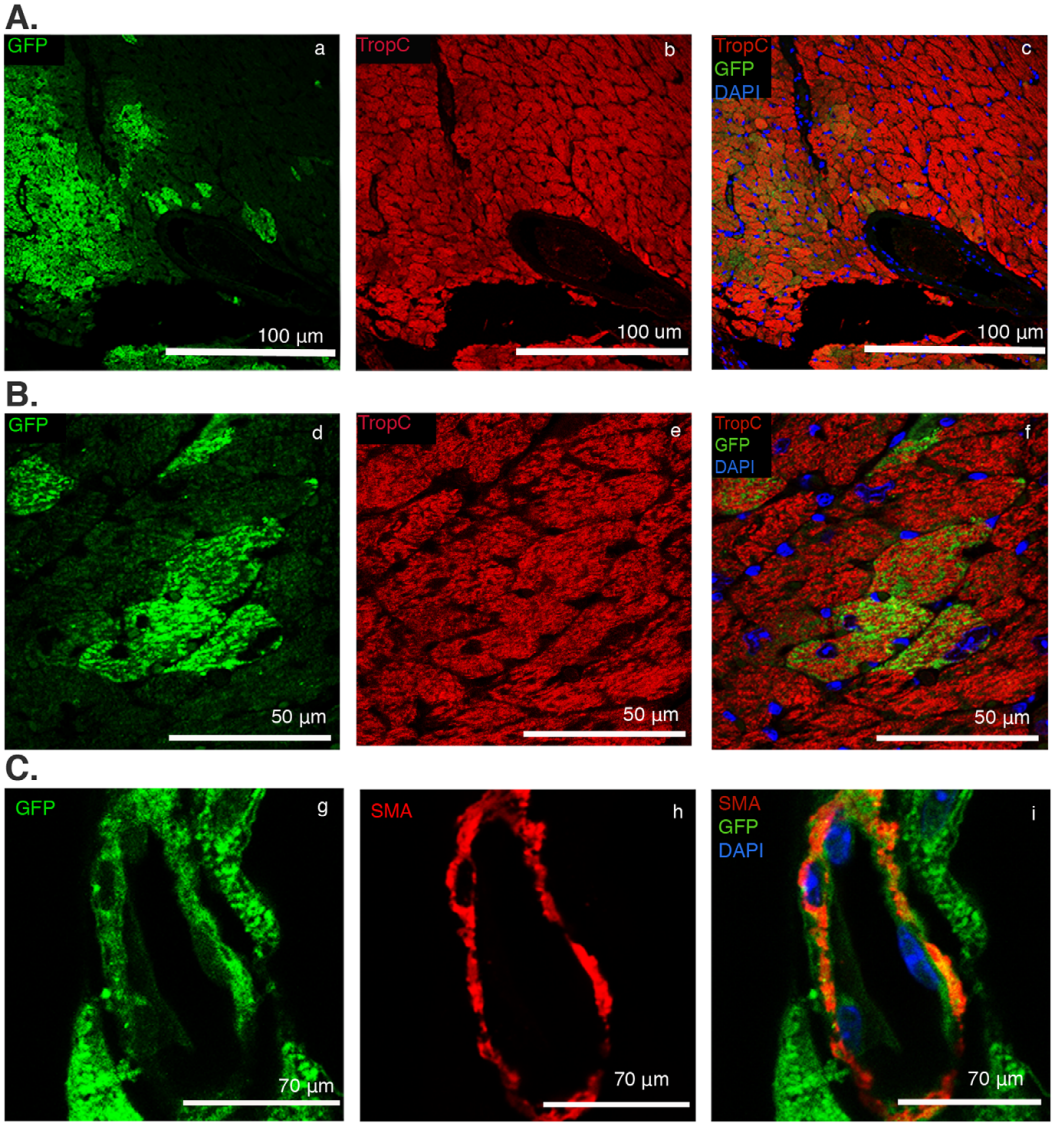
C57/BL6 iPSC-derived GFP^+^ Flt1^+^/Flt4^+^ CPCs transplanted into strain-matched hearts engraft and robustly differentiate into CMs and smooth muscle cells. Post-injection immunofluorescence imaging of GFP^+^ Flt1^+^/Flt4^+^ CPCs transplanted into the left ventricle of C57/BL6 mice demonstrates *in vivo* engraftment and robust differentiation into CMs and smooth muscle cells. (A & B) Immunostaining of GFP^+^ Flt1^+^/Flt4^+^ injected myocardial sections for CMs: (a,d) single channels for GFP and (b,e) single channels for TropC with (c,f) merged images. (C) Immunostaining of GFP^+^ Flt1^+^/Flt4^+^ injected myocardial sections for smooth muscle cells: (g) single channel for GFP and (h) α-SMA along with (i) merged images.

**Figure 6:**
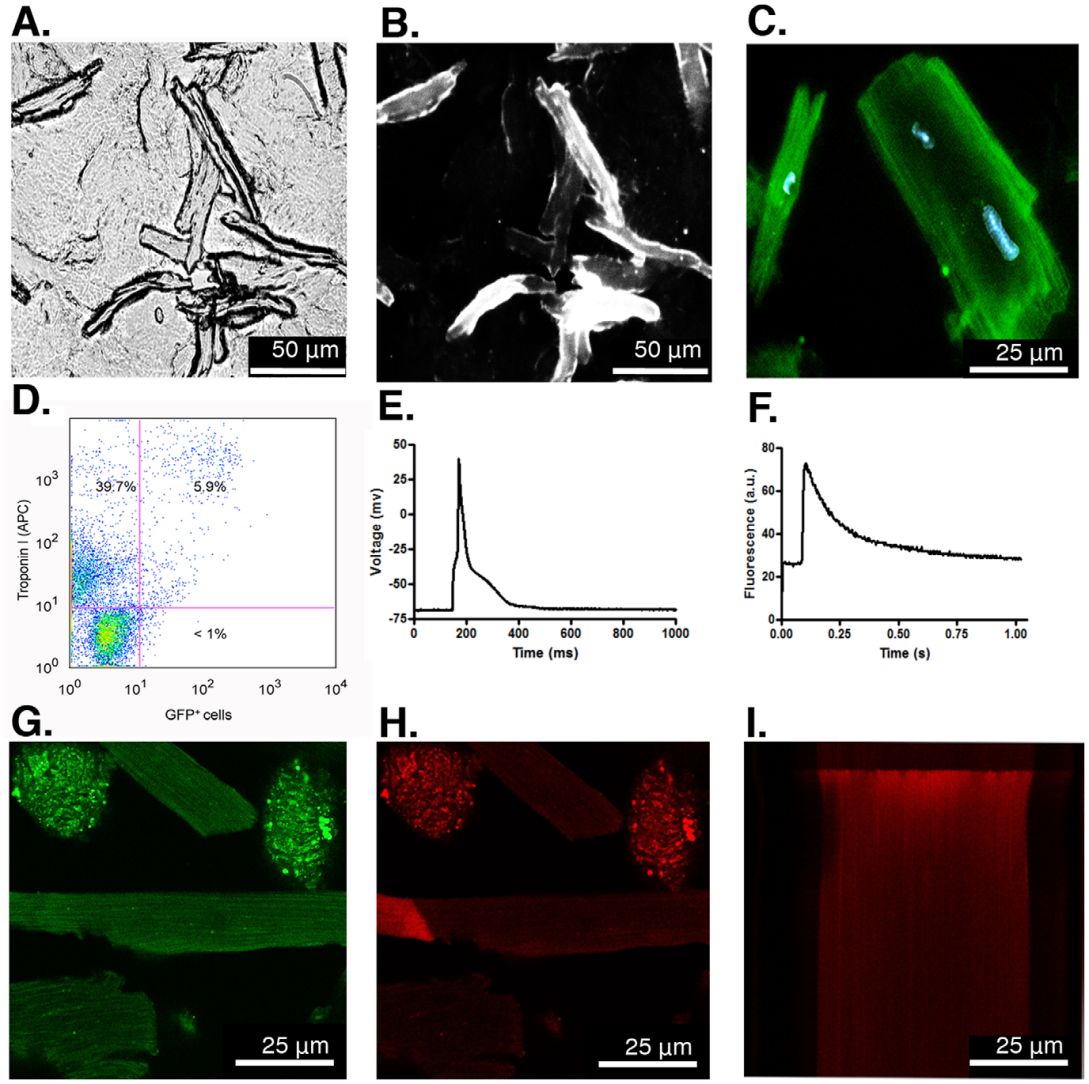
Transplanted iPSC-derived Flt1^+^/Flt4^+^ CPCs differentiate into morphologically and electrophysiologically mature CMs. (A) 10X phase contrast imaging of live, dissociated single CMs post-transplantation of GFP^+^ Flt1^+^/Flt4^+^ CPCs into the LV. (B) Immunofluorescence imaging of the same field of live cells confirms GFP^+^ mature CMs derived from transplanted CPCs. (C) 40X image of GFP^+^ CMs counterstained using DAPI. (D) FACS analysis of dissociated cells from transplanted hearts demonstrates that ∼6% of Troponin I^+^ CMs are GFP^+^ labeled using a GFP antibody. (E) Action potential and (F) fluorescence transient recorded simultaneously from a single GFP^+^ Flt1^+^/Flt4^+^-derived CM loaded with the Ca^2+^ indicator rhod-2-AM and paced at 0.2 Hz. (G) Confocal image of live single GFP^+^ Flt1^+^/Flt4^+^-derived CMs post-enzymatic digestion of mouse heart post-transplant (63X). (H) Live 2D Ca^2+^ imaging of the same GFP^+^ CM shown in G which was also loaded with Ca^2+^ indicator rhod-2-AM. The bright area on the left shows the beginning of a spontaneous Ca^2+^ wave. (I) Line scan image from the same CM during depolarization evoked by field stimulation. The synchronous onset of the Ca^2+^ transient and rapid increase in fluorescence indicate electrically triggered Ca^2+^ release. Fluorescence intensities are displayed in arbitrary units (a.u.) and action potential in millivolt (mV). Scale bars equal 25 µm.

One limitation of many cell types used for cardiac cell therapy is their limited capacity for engraftment and differentiation to mature CMs. To assess the ability of Flt1^+^/Flt4^+^ CPCs to engraft and differentiate *in vivo*, we isolated Flt1^+^/Flt4^+^ CPCs from GFP^+^ mouse iPSCs and transplanted them into normal hearts of strain-matched mice. iPSC-derived GFP^+^ Flt1^+^/Flt4^+^ CPCs were suspended in a pro-survival media and injected into the anterior wall of the left ventricle of adult mice. Examination of the transplanted myocardium after 28 days revealed robust engraftment and differentiation of GFP^+^ Flt1^+^/Flt4^+^ CPCs into Troponin C-expressing mature CMs (Fig. 5A and 5B) and α-smooth muscle actin-expressing SMCs (Fig. 5C). In contrast to the *in vitro* studies, we were not able to detect differentiation into ECs. Histological assessment for an immune reaction against injected CPCs revealed no evidence of infiltration of CD45^+^ lymphocytes into the transplanted region (Figure S3). Digestion and single cell analysis of the GFP^+^ Flt1^+^/Flt4^+^ CPC-transplanted hearts revealed mature, adult-appearing GFP^+^ CMs (Fig. 6A, 6B and 6C). Since many of the GFP^+^ CMs were mononucleated this could not be explained by fusion of transplanted CPCs with existing CMs (Fig. 6C). FACS analysis of single cell suspensions demonstrated that ∼6% of the total cell population were GFP^+^/Troponin I^+^ representing CMs derived from the transplanted CPCs (Fig. 6D). To confirm that the GFP^+^ Flt1^+^/Flt4^+^-derived CMs were also electrophysiologically mature, we loaded them with the calcium indicator rhod-2 AM. GFP^+^ CMs (Fig. 6G) could be externally paced at frequencies of 0.2 Hz to generate characteristic action potentials (Fig. 6E), Ca^2+^ transients and cell shortening indicative of electrically functional CMs (Fig. 6F, 6H, 6I). The synchronous onset and rapid upstroke of the Ca^2+^ transient indicate electrically triggered Ca^2+^ release typical of adult CMs (Fig. 6F). Thus, iPSC-derived CPCs have the potential to engraft into the adult myocardium and robustly differentiate into CMs with phenotypic and electrophysiologic characteristics of adult CMs.

## Discussion

To realize the true potential of cardiovascular regenerative therapy, it will be necessary to identify a source of cardiac progenitor cells that can be easily isolated from a renewable source without immunologic or oncogenic concerns. Multipotent CPCs derived from differentiating iPSCs based on well-defined surface markers and differentiation conditions would potentially address this gap in cardiac cell therapy. Unlike the hematopoietic system where the hierarchy of differentiation of progenitors is clearly delineated and the surface markers well-defined [21], there is no universally agreed upon developmental hierarchy for cardiovascular progenitors as defined by surface markers. Multiple groups have described different progenitors with lineage tracing and gene-targeting studies as well as FACS analysis, all based on intracellular transcription factors [22], [23], [24]. To date, studies identifying cell surface markers for CPCs have been based mostly on ESC studies evaluating different cell surface markers and their enrichment for CPC transcription factors such as Nkx2.5 [25] and Isl1 [20]. This study is the first to our knowledge to identify and functionally validate specific cell surface markers for endogenous multipotent Isl1^+^/Nkx2.5^+^ CPCs and to recapitulate this CPC in differentiating iPSCs. The novel surface marker combination of Flt1 and Flt4 was identified by our microarray analysis of the heterogeneous, ESC-derived Flk1^+^ cell population [13]. These markers identify and enrich for a true CPC and are more specific than other cell surface marker combinations that have been described to identify endogenous Isl1^+^ CPCs in the embryonic heart as demonstrated by our FACS analysis (Table 1). It is very likely, given the hierarchy that has been proposed in cardiovascular cell development, that cardiovascular progenitors will express a unique cell surface phenotype as they develop and enrich to varying extents for CPCs [18], [25], [26]. When isolated, Flt1^+^/Flt4^+^ CPCs from both endogenous and iPSC sources expressed the cardiogenic transcription factors Isl1 [23] and Nkx2.5 [25] and were capable of differentiating into all three cardiovascular cell types. Furthermore, Flt1^+^/Flt4^+^ CPCs demonstrated more robust *in vitro* differentiation into CMs compared to Flk1^+^ cells. Not surprisingly, the more robust differentiation potential of Flt1^+^/Flt4^+^ CPCs to CMs as compared to Flk1^+^ cells is likely due to the fact that the Flt1^+^/Flt4^+^ cell population represents a more homogeneous CPC population compared to the heterogeneous population identified by Flk1. Moreover, the iPSC-derived Flt1^−/^Flt4^−^ cells showed no *in vitro* differentiation potential into CMs, confirming that the combination of Flt1^+^/Flt4^+^ enriched for the majority of CPCs from differentiating iPSCs.

Although not a cardiac-specific marker, Isl1 has been identified by many groups to developmentally label cardiovascular progenitors at early post-gastrulation stages of mouse development that will give rise to the second heart field, which is primarily responsible for generating the right ventricle (RV), atria and outflow tracts, and to partially contribute to the formation of the conduction system [22], [23]. Additionally, intersectional fate mapping has determined that in the embryonic heart, Isl1^+^ cells label, along with the second heart field forming cells, cells derived from the cardiac neural crest and the cardiac ganglia [27], [28], [29]. Furthermore, Weinberger et al. demonstrated in the adult murine heart between 1 month and 18 months that Isl1 labels cells of the sino-atrium; however, there was no evidence of Isl1^+^ cells post infarct [30]. Our attempts to identify Isl1^+^ cells in the adult myocardium by flow cytometry and immunofluorescence have been unsuccessful. Our staining of embryonic hearts identified two subpopulations of Isl1^+^ cells based on their expression of neurological markers: those positive for both neuronal marker Map2 and the peripheral ganglia marker NEFH, and those positive only for Map2. We believe these Map2^+^ cells represent neural crest-derived Isl1^+^ cells which, along with those found earlier in development, can act as CPCs in agreement with recent work [31], [32].

The use of Isl1 as a marker to identify progenitors for cell transplantation has been criticized because it theoretically identifies a CPC that will give rise to a right ventricular CM, which might not function physiologically the same as a left ventricular CM. However, there is little evidence to support this assertion and these same endogenous Isl1^+^ progenitors also contribute to the left ventricle [8], [26], [33]. Additionally, an Isl1^+^ epicardial cardiac progenitor has been identified that generates CMs in the LV [34]. We demonstrate that iPSC-derived Flt1^+^/Flt4^+^ CPCs, when transplanted into the LV of strain-matched mice, engraft and preferentially differentiate into CMs that morphologically and electrophysiologically resemble adult left ventricular CMs (Fig. 6). Functionally, these Flt1^+^/Flt4^+^ CPCs demonstrate a robust *in vitro* and *in vivo* cardiovascular differentiation potential, which is the main aim of this study to identify specific surface markers to isolate iPSC-derived CPCs for potential regenerative therapies.

Although, Flt1^+^/Flt4^+^ CPCs demonstrated a clonal trilineage differentiation potential *in vitro*, we were able to detect potent cardiac differentiation *in vivo* as well as smooth muscle differentiation, but no demonstrable endothelial cell differentiation. As well, Flt1^+^/Flt4^+^ CPCs consistently formed beating colonies and CMs *in vitro*, which appeared to be more fetal in phenotype as seen on immunofluorescence staining (Fig. 4Ad; Figure S2), once transplanted *in vivo*, they formed CMs with a phenotype and electrophysiological properties typical of adult CMs (Fig. 6). The reason for this discrepancy is unclear, but external cues from the local microenvironment play a critical role in determining cell fate [16] and, *a priori*, intramyocardial injections would be predicted to favor cardiomyocyte differentiation. The local extracellular matrix along with electromechanical stimulation *in vivo* may play a role in evolving CMs to a more mature phenotype. Thus, the ability to deliver a well-characterized progenitor population derived from iPSCs capable of integration and robust cardiomyocyte differentiation could be a significant advance for regenerative cardiac cell therapies.

Both the study and clinical application of the various cardiovascular progenitor cell types has been limited by their scarcity and lack of appropriate conditions to expand them in an undifferentiated state. Progenitor cells are notoriously difficult to maintain in culture; for example, suitable conditions do not exist to culture hematopoietic stem cells *in vitro* despite the fact they were identified over two decades ago [21]. Here, we describe a novel approach to expand rare cardiovascular progenitors while maintaining their trilineage potential in a feeder free manner by using two small molecules IQ-1, a selective β-catenin inhibitor that has been shown to maintain pluripotency in ESCs [14], and Thiazovivn, a ROCK inhibitor, which has been shown to prevent anoikis and improve survival of single cell ESCs [20]. The use of IQ-1 differs from other approaches targeted at inhibiting Wnt/β-catenin signals because instead of globally blocking all Wnt/β-catenin signals, IQ-1 selectively inhibits the subset of β-catenin signaling mediated by its interaction with p300, which is pro-differentiation. We demonstrate that the combination of these small molecules maintains CPCs in their mutlipotent state and allows expansion to generate sufficient numbers to allow their characterization and transplantation *in vivo* (Fig. 4).

There is increasing awareness that although cell therapy for ischemic heart disease with adult stem cells may be safe and results in a modest improvement in ventricular function, current clinical approaches have largely failed at achieving significant myogenesis [35]. Any modest effects seen may be transient and secondary to paracrine effects of transplanted cells rather than true tissue regeneration. This has resulted in calls for the development of cell therapy approaches with cell types capable of cardiomyogenic regeneration, including iPSC-derived cells, which could result in true myocardial regeneration [4]. While the clinical applicability of early iPSCs has been questioned because of a potential oncogenic risk related to the genes used for reprogramming and requirement for genomic integration, safer more efficient non-viral methods for reprogramming of human somatic cells have now been described [36], [37]. It is likely that FDA-approvable human iPSCs lines will be generated in the near future. The use of more differentiated derivatives should also reduce any risks. However, the relative efficacy of using CPCs versus more differentiated derivatives such as iPSC-derived CMs for cell therapy will need to be determined empirically. Here, we have focused our efforts on iPSC-derived CPCs because of their potential for more complete regeneration secondary to their multipotency and possible improved safety profile as compared to transplanting undifferentiated pluripotent cells such iPSCs or ECSs. We have shown that mouse iPSC-derived CPCs can be cloned, which might allow the development of a homogeneous cell product that could be delivered devoid of any undifferentiated iPSCs contaminants. Selective manipulation of β-catenin signaling in Flt1^+^/Flt4^+^ CPCs by using the small molecule IQ-1, as well as the use of the ROCK inhibitor small molecule Thiazovivn, permitted their feeder-free expansion *in vitro*, providing for the first time a significant source of clonally-derived and expanded CPCs, free of feeder cells, for study in *in vivo* models. As well, *in vivo* transplantation demonstrated that iPSC-derived Flt1^+^/Flt4^+^ CPCs have the capacity to engraft into the native tissue with robust CM differentiation. A recent concern with iPSC-based regenerative therapies is the possibility of immunogenicity even in immunologically matched iPSCs [38]. The reprogramming process to derive iPSCs, whether it involves retroviral or non-integrative viral-free approaches to over-express reprogramming transcription factors, may lead to abnormal expression of tissue-specific or tumor-specific surface antigens in some iPSC-derived cells [38]. This in turn can induce a T-cell-dependent immune response post transplantation of iPSC-derived progenitors, potentially leading to the destruction of the transplanted iPSCs cells [38]. We did not observe any immune response generated by transplantation of iPSC-derived CPCs as we demonstrate robust survival and engraftment of the iPSC-derived CPCs (Figure S3). This could be related to a number of factors. At least part of the observed immunological response previously reported could be related to embryonic antigens present on the iPSCs, not antigens related to the reprogramming. We transplanted more differentiated lineage-committed CPCs, which may be necessary when transplanting into adult tissues. As well, the pro-survival injection media we used contains the immunosuppressant cyclosporine. Although cyclosporine may not be powerful enough to suppress the immune response to Human Leukocyte Antigen (HLA)-mismatched ESCs [39], [40], [41], it may be sufficient to overcome any immunological response to iPSC-derived CPCs. Regardless, the techniques described here will facilitate the study of iPSC-derived CPCs and potentially the generation of patient-specific CPC lines, providing a foundation for future clinical application.

## Supporting Information

Figure S1 **Isl1^+^ cell staining for the neuronal marker Map2 and ganglia marker NEFH.** (A) Staining of mouse E15.5 hearts for Microtubule-associated protein 2 (Map2), a pan-neuronal marker, revealed that most Isl1^+^ cells in the clusters were positive for Map2. (B) Staining for Neurofilament, Heavy Polypeptide (NEFH), a marker of ganglia, demonstrated that most Isl1^+^ cells within the core of the cluster were positive for NEFH. Arrows indicate Isl1^+^/NEFH^−^ cells on the periphery of the cluster. 20X magnification. Cell nuclei are counterstained using 4'-6-diamidino-2-phenylindole (DAPI) in blue.
(TIF)

Figure S2 **Clonally expanded Flt1+/Flt4+ CPCs differentiate into beating colonies with MF-20 (in red) striated cardiomyocytes seen at 40X.** A) 10X, B) 20X, C) 40X. Cell nuclei are counterstained using 4'-6-diamidino-2-phenylindole (DAPI) in blue.
(TIF)

Figure S3 **Mouse iPSC-derived CPCs do not illicit an immune response** ***in vivo*** **post transplant.** (A) No evidence of a cellular immune reaction to differentiated iPSC-derived GFP^+^ CPCs (green) twenty-eight days post transplant into left ventricles of strain matched hearts in pro-survival media as assayed by staining for GFP^+^ cells and CD45^+^ lymphocytes (red). 10X magnification. (B) CD45 positive control in a wild type mouse 21 days post infarction of Left Anterior Descending (LAD) artery reveals infiltration of CD45^+^ lymphocytes in infarct zone area. 10X magnification. Cell nuclei are counterstained using 4'-6-diamidino-2-phenylindole (DAPI) in blue.
(TIF)

Methods S1 **Supplementary Methods.**
(DOC)

Movie S1 **Clonally expanded CPCs differentiate into beating cardiac myocytes.**
(MOV)

Table S1 **Primary and secondary antibodies.**
(DOC)
