# Correction: Characterization and Therapeutic Potential of Induced Pluripotent Stem Cell-Derived Cardiovascular Progenitor Cells

**DOI:** 10.1371/annotation/649fb8ef-a4c7-4ee7-b382-e1657b9aa07e

**Published:** 2012-10-15

**Authors:** Ali Nsair, Katja Schenke-Layland, Ben Van Handel, Denis Evseenko, Michael Kahn, Peng Zhao, Joseph Mendelis, Sanaz Heydarkhan, Obina Awaji, Miriam Vottler, Susanne Geist, Jennifer Chyu, Nuria Gago-Lopez, Gay M. Crooks, Kathrin Plath, Josh Goldhaber, Hanna K. A. Mikkola, W. Robb MacLellan

The published funding statement was incorrect. The correct funding is:

This work was supported by the NIH (R01-HL70748 to W.R.M.), Ruth Kirschstein National Research Service Award (GM07185 and T32 HL69766 to B.V.H), the Cardiovascular Development Funds and Alberta Heritage Foundation for Medical Research Fellowship (CDF and AHFMR to A.N), the BMBF (0316059), Fraunhofer-Gesellschaft Internal programs (Attract 692263) as well as the Ministry of Science, Research and the Arts of Baden-Württemberg (33-729.55-3/214) (to K.S.-L.). The funders had no role in study design, data collection and analysis, decision to publish, or preparation of the manuscript.

