# Supplementary figures and images for: Characterization and Therapeutic Potential of Induced Pluripotent Stem Cell-Derived Cardiovascular Progenitor Cells
Source: PLoS One. 2012 Oct 9;7(10):e45603. doi: 10.1371/journal.pone.0045603 (PMC3467279; doi:10.1371/journal.pone.0045603)

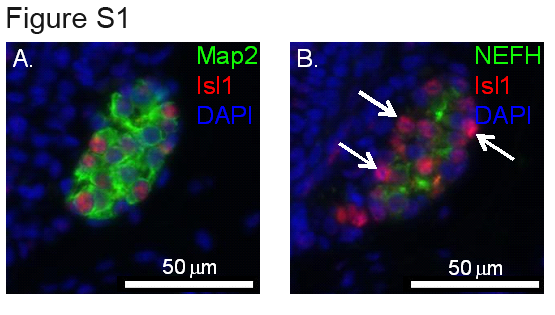

Supplement: Figure S1 — Isl1+ cell staining for the neuronal marker Map2 and ganglia marker NEFH. (A) Staining of mouse E15.5 hearts for Microtubule-associated protein 2 (Map2), a pan-neuronal marker, revealed that most Isl1+ cells in the clusters were positive for Map2. (B) Staining for Neurofilament, Heavy Polypeptide (NEFH), a marker of ganglia, demonstrated that most Isl1+ cells within the core of the cluster were positive for NEFH. Arrows indicate Isl1+/NEFH− cells on the periphery of the cluster. 20X magnification. Cell nuclei are counterstained using 4'-6-diamidino-2-phenylindole (DAPI) in blue. (TIF) [file pone.0045603.s001.tif]

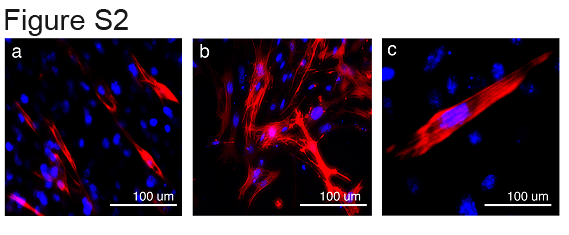

Supplement: Figure S2 — Clonally expanded Flt1+/Flt4+ CPCs differentiate into beating colonies with MF-20 (in red) striated cardiomyocytes seen at 40X. A) 10X, B) 20X, C) 40X. Cell nuclei are counterstained using 4'-6-diamidino-2-phenylindole (DAPI) in blue. (TIF) [file pone.0045603.s002.tif]

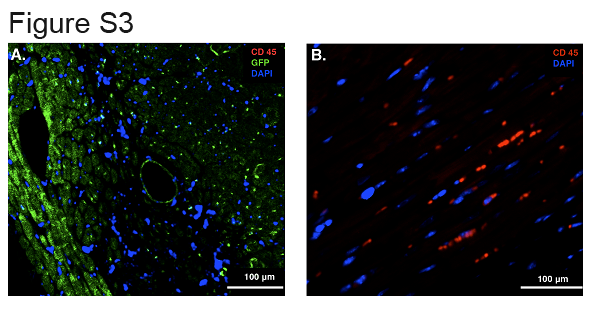

Supplement: Figure S3 — Mouse iPSC-derived CPCs do not illicit an immune response in vivo post transplant. (A) No evidence of a cellular immune reaction to differentiated iPSC-derived GFP+ CPCs (green) twenty-eight days post transplant into left ventricles of strain matched hearts in pro-survival media as assayed by staining for GFP+ cells and CD45+ lymphocytes (red). 10X magnification. (B) CD45 positive control in a wild type mouse 21 days post infarction of Left Anterior Descending (LAD) artery reveals infiltration of CD45+ lymphocytes in infarct zone area. 10X magnification. Cell nuclei are counterstained using 4'-6-diamidino-2-phenylindole (DAPI) in blue. (TIF) [file pone.0045603.s003.tif]
