# Supplementary material for: Characterization and Therapeutic Potential of Induced Pluripotent Stem Cell-Derived Cardiovascular Progenitor Cells
Source: PLoS One. 2012 Oct 9;7(10):e45603. doi: 10.1371/journal.pone.0045603 (PMC3467279; doi:10.1371/journal.pone.0045603)
Supplement: Methods S1 — Supplementary Methods. (DOC) [file pone.0045603.s004.doc]

**Microarray Analysis**

RNA samples from undifferentiated mouse embryonic stem cells (mESC), as well as mESC-derived Flk1^-^ cells and Flk1^+^ progenitor cells [13] were analyzed at the UCLA Illumina Microarray Laboratory. Biotinylated cRNA was prepared using the Illumina RNA Amplification Kit (Ambion Inc., Austin, TX, http://www.ambion.com) starting with 100 ng of total RNA. Samples were purified and used for hybridization on a Sentrix MouseRef-8 Expression BeadChip System (Illumina Inc., San Diego, http://www.illumina.com) containing approximately 24,000 reference-sequence-based probe sequences per array. Scanning was performed according to the Illumina BeadStation 500 manual. Microarray raw data were analyzed using BeadStudio software version 1.5.1.3. that was provided by the manufacturer. Differential expression analysis was selected to quantify gene expression intensity values as well as to determine changes of the gene expression levels between undifferentiated ES cells (reference group) and ColIV-differentiated Flk1^-^ and Flk1^+^ cells. To filter out nonspecific signal intensities, local background subtraction was performed. Only genes with intensities >0.99 were selected for analysis. A differential score of.0 demonstrated that gene expression from ColIV-differentiated ESC had changed significantly when compared with genes of undifferentiated mouse ESC.

**Gene expression analysis**

Total RNA was extracted from mouse hearts, murine ES cells, as well as from mouse undifferentiated and differentiated Flt1^+^/Flt4^+^ cells. Semi-quantitative PCR was performed as previously described [13]. All mouse primer sequences have been previously published [13]. Primer sets specific for mouse Flt1 and Flt4 were purchased from R&D Systems. Mouse glyceraldehyde-3-phosphate dehydrogenase (Gapdh) primer sets were obtained from Qiagen (QuantiTect Primer Assay). All commercially available primer sets were used following the manufacture’s instructions.

**Immunohistochemistry**

Slides were deparaffinized and rehydrated through a xylene/alcohol gradient and blocked in blocking buffer (5% horse serum, 0.05% Tween 20 in PBS) for 15 minutes at room temperature. Primary antibodies were applied for 1 hour and detected with biotinylated secondary antibodies. Secondary antibodies were detected using the ABC system (Vector) according to the manufacturer’s instructions. For mouse primary antibodies on mouse tissues, the M.O.M. kit (BMK-2202, Vector) was used as directed. To counterstain cell nuclei, 4'-6-diamidino-2-phenylindole (DAPI, Sigma) was used.

**Immunofluorescence staining and confocal microscopy**

Unstained sections were deparaffinized and rehydrated through a xylene/alcohol gradient. Antigen retrieval was performed at 95°C for 30 minutes each sequentially in 10mM Tris, 1 mM EDTA, 0.05% Tween 20 solution, pH 9.0 followed by 10 mM citrate solution in PBS, pH 6.0. After cooling, endogenous peroxidases were quenched in a 0.9% solution of H_2_O_2_ in methanol for 20 minutes at room temperature. Slides were permeabilized with 1% Triton X-100 in PBS for one hour, then blocked in tyramide blocking solution (Invitrogen) plus 1% Triton X-100 (TBST) for one hour. Slides were incubated with primary antibodies overnight at 4°C, washed with 0.1% Tween in PBS (PBST) and incubated with biotinylated secondary antibodies (1:500, Vector) in TBST for 30 minutes at room temperature. Staining with the corresponding IgG controls for each one of the antibodies (Santa Cruz), and staining without primary antibodies (background detection) served as controls. All samples were then washed with PBST (3x) and incubated with streptavidin-HRP (Invitrogen, 1:500 in TBST) for 30 minutes at room temperature, followed by washing in PBST (3x) and tyramide biotin-XX amplification for 7.5 minutes as per manufacturer’s instructions (Invitrogen). Slides were then washed in PBST and incubated with ABC Elite (Vector Labs) for 30 minutes at room temperature. After washing in PBST, all slides were incubated with tyramide-fluorophore for 7.5 minutes prepared as directed (Invitrogen), washed in PBS and then incubated with 0.05M HCl for 20 minutes at room temperature to quench peroxidases. Slides were washed 3x in PBST, then blocked and incubated with primary antibodies as above. After the final tyramide-fluorophore step, slides were washed in PBS, incubated with DAPI solution (5 mg/ml in PBS, Sigma) for 5 minutes at room temperature, washed 3x with PBS and mounted in ProLong Gold mounting medium (Molecular Probes) and stored at 4°C prior imaging. Fluorescence images were acquired using a confocal TCS SP2 AOBS laser-scanning microscope system (Leica Microsystems Inc.). Images were processed with Adobe Photoshop CS3 (Adobe Systems Inc.).

**Primary and secondary antibodies**

Primary antibodies used in this study include (I) rabbit polyclonal antibodies: anti-alpha smooth muscle actin (αSMA; ab5694 (1:400); abcam, Cambridge, MA, [www.abcam.com](http://www.abcam.com/)); anti-Flt1 (ab2350 (IF 1:1000, FACS 1:50); abcam); anti-Flt4 (ab27278 (IF 1:1000, FACS 1:50); abcam); anti-Troponin C (sc-48347 (1:50); Santa Cruz Biotechnology, Inc., Santa Cruz, CA, [www.scbt.com/](http://www.scbt.com/)); Nkx2.5 (ab22611 (1:250); abcam); (II) mouse monoclonal antibodies: anti-Flk1 (sc-6251; (1:1500); Santa Cruz); anti-Isl1 (40-206, (IF and FACS, 1:250); Developmental Studies Hybridoma Bank (DSHB), Iowa City, IA, http://dshb.biology.uiowa.edu); (III) a rat monoclonal antibody: anti-Pecam1 (CD31, 550274 (1:50); BD Pharmingen, San Diego, CA, [www.bdbiosciences.com/pharmingen](http://www.bdbiosciences.com/pharmingen)). Secondary antibodies included Alexa Fluor 488-, Alexa Fluor 594-, Alexa Fluor 647-conjugated goat-anti mouse IgG (H+L); goat-anti rabbit IgG (H+L); goat-anti rat IgG (H+L) and goat-anti mouse IgM (H+L) (1:250; all from Molecular Probes, Eugene, OR, probes.invitrogen.com).

**Flow cytometry analysis**

Cells were fixed in 1% PFA for 15 minutes on ice, quenched with glycine and stained with extracellular primary antibodies conjugated with Zenon kits (Invitrogen) used as per manufacturer’s instructions. Cells were post-fixed for 5 minutes as above, permeabilized using PermWash buffer (BD Biosciences) and stained with Isl1 antibody conjugated using a Zenon AlexFluor 647 kit (Invitrogen). Cells were analyzed immediately on a BD LSR II cytometer (BD Biosciences). Data analysis was performed using FCS files that were exported and analyzed using the FlowJo 8.6.3 software (Tree Star Inc.).

**Isolation and expansion of endogenous CPCs**

Murine hearts dissected from E15.5 embryos (CF1- mice; Charles River, Inc.) were mechanically dissociated by mincing with scalpels and then subjected to enzymatic digestion in 10 ml of enzyme solution per gram of tissue [2.5 U dispase (Invitrogen), 5% ES cell-qualified fetal calf serum (ES-FBS; Invitrogen), 10 mg collagenase (Sigma), 1% amphotericin B (250 µg/mL, Invitrogen), 1% penicillin-streptomycin (10,000 U/ml-10,000 µg/ml, Gibco) and 0.01% Ciprofloxacin HCl (Sigma)] for 30 minutes with agitation at 37°C. The solution was then resuspended for 3 times through a syringe with an 18G needle, followed by treatment with DNase I (0.075 mg/g of tissue; Sigma) for an additional 30 minutes. Samples were then filtered through 70-µm mesh prior to fluorescence-activated cell sorting (FACS) or FACS analysis. For culturing cells, the murine cells were maintained on mitomycin-C-treated, primary mouse embryonic fibroblasts (MEF) in IQ1-supplemented medium [1:1:1 alpha-Minimum Essential Medium (Invitrogen), endothelial growth medium (EGM) and smooth muscle growth medium SMGM (both Lonza); supplemented with 4 µg/ml IQ1. All cells were cultured at 37°C, 5% CO_2_. After cell expansion (~6-10 days), murine cells were trypsinized and the Flt1^+^/Flt4^+^ cells were isolated by indirect magnetic cell sorting (MACS) (Stem Cell Technologies) using conjugated Flt1 and Flt4 antibodies (Abcam) and prior exposure to *in vitro* differentiation assays. Cells were recovered post MACS sorting and plated onto fibronectin coated culture plates (BD bioscience).

**Differentiation assays**

For *in vitro* differentiation assays, murine MACS-isolated Flt1/Flt4 double positive cells were plated on fibronectin-coated culture slides (BD Bioscience Discovery Labware) in either alpha-MEM (cardiac differentiation), PDGF-BB medium [SMGM, supplemented with 10 ng/ml platelet-derived growth factor-BB (PDGF-BB; R&D Systems Inc.)] (smooth muscle differentiation), or VEGF medium [EGM, supplemented with 50 ng/ml vascular endothelial growth factor (VEGF; R&D Systems Inc.)] (EC differentiation) as described before [13].

**Clonal expansion and differentiation of CPCs**

Skin fibroblasts from C57/BL6 mice were reprogrammed as described4 and stably transfected with a constitutively expressed green fluorescence protein (GFP). These GFP^+^ mouse iPSCs were cultured in standard conditions on Mitomycin-C inactivated feeder layers in LIF media as described before [13]. Post expansion, GFP^+^ mouse iPSCs were dissociated using Accutase (Sigma) and plated on Collagen IV coated plates (BD bioscience) and cultured for four days in α-MEM media to induce differentiation of mouse iPSCs into CPCs as previously demonstrated [13]. At day four, cells were dissociated with Accutase (Invitrogen) and single cell FACS sorting was performed for Flt1^+^/Flt4^+^ population using conjugated Flt1 and Flt4 antibodies (Santa Cruz). FACS sorting was performed on ARIA II Flow Cytometer at UCLA core facility. Post sorting, Flt1^+^/Flt4^+^ cells were recovered in CPC clonal media prepared using feeder free media ESGRO (Millipore) supplemented with IQ-1 (Calbiochem) at 4 µg/ml and ROCK inhibitor Thiazovivin (Stemgent) at 2 μM to enhance survival, and expanded on fibronectin-coated plates (BD Bioscience Discovery Labware). Post expansion for four weeks, single clones were isolated and dissociated into single cell suspensions and re-plated onto fibronectin-coated culture slides (BD Bioscience Discovery Labware) in either alpha-MEM (cardiac differentiation), PDGF-BB medium [SMGM, supplemented with 10 ng/ml platelet-derived growth factor-BB (PDGF-BB; R&D Systems Inc.)] (smooth muscle differentiation, or VEGF medium [EGM, supplemented with 50 ng/ml vascular endothelial growth factor (VEGF; R&D Systems Inc.)](EC differentiation) as described before [13]. Once spontaneously beating colonies were observed fourteen days post differentiation, cells were fixed with 1% paraformaldehyde for immunostaining.

***In vivo* transplant of Cardiac Progenitor Cells**

GFP^+^ mouse iPSCs were used to derive GFP^+^ Flt1^+^/Flt4^+^ CPCs. As described above, GFP^+^ mouse iPSCs were expanded and differentiated into Flt1^+^/Flt4^+^ CPCs and isolated with FACS sorting. Post FACS sort, Flt1^+^/Flt4^+^ CPCs were recovered in CPC pro-survival media prior to transplant into strain matched C57 mice. CPC pro-survival media was prepared with ESGRO media enriched with pro-survival growth factors [15]: growth factor–reduced Matrigel (BD bioscience) 50% (vol/vol), supplemented with ZVAD at 100 μM (benzyloxycarbonyl-Val-Ala-Asp(O-methyl)-fluoromethyl ketone, Calbiochem), Bcl-X_L_ BH4 at 50 nM (Calbiochem), Cyclosporine A (200 nM, Wako Pure Chemicals), IGF-1 at 100 ng/ml (Peprotech), and pinacidil at 50 μM (Sigma). Flt1^+^/Flt4^+^ CPCs were recovered overnight in CPC pro-survival media [15] at 4°C. Flt1^+^/Flt4^+^ CPCs were suspended at 50,000 cells per 50 μl in CPC pro-survival media and injected into the anterior wall of the left ventricle at 10 μl per injection site for a total of 5 injections. Sham injections with pro-survival media were performed on control mice as well. For *in vivo* transplant, mice were anesthetized with general anesthesia (sodium pentobarbital, 60 mg/kg, i.p. with maintenance doses (15mg/Kg) given as needed to maintain deep surgical anesthesia). Mice were intubated with endotracheal tubing via oral intubation. The tubing was connected to a positive pressure respirator. Survival surgery consisted of a thoracotomy, which is required to gain access to the left cardiac ventricle. Under mechanical ventilation, the chest was opened through a central thoracotomy and the heart was exposed. Post injection, the chest was closed in layers (absorbable sutures used for the muscle and nylon/prolene for the skin), and the animals recovered under close observation. 22 days post injection, the animals were sacrificed, hearts were harvested, fixed in 10% Formalin for 12 hours, and after which transferred to 70% ethanol for paraffin sections for histology. Immunofluorescence labeling and imaging was performed as described above.

**Adult cardiomyocyte isolation and simultaneous recording of Ca^2+^ transients and action potentials in GFP^+^ Flt1^+^/Flt4^+^-derived CMs from transplanted CPCs**

We isolated murine ventricular cardiomyocytes using the technique we described previously (#see Reuter reference in the comments#). Adult mice received an intraperitoneal injection with 400μl of heparin (5,000 units/ml) twenty minutes prior to euthanizing them. We then anesthetized the mice with isoflurane and excised their hearts via a thoracotomy. We enzymatically isolated single ventricular myocytes using retrograde aortic perfusion of collagenase (1 mg/ml, Type II collagenase; Gibco BRL) and protease (0.1 mg/ml, Type XIV protease; Sigma-Aldrich) at 37^o^C. The isolated cells were washed three times and resuspended at room temperature in modified Tyrode’s solution, containing (in mmol/L): 136 NaCl, 5.4 KCl, 10 HEPES, 1.0 MgCl2, 0.33 NaH2PO4, 0.5 CaCl2, 10 glucose, (pH 7.4).

To load isolated CMs with the cell-permeant Ca-sensitive indicator rhod-2 AM (Invitrogen, R1245MP) we prepared a 2 mM stock solution: 50ug rhod-2 AM in 22.25 μl DMSO. We then added 7.5 μl of 2 mM rhod-2AM stock and 1 μl 0.02% Pluronic F-127 (dissolved in DMSO, Molecular Probes) to 1000 µl of cell suspension (final rhod-2 AM concentration, 15 µM). Cells were washed three times over a 45 minute period and resuspended at room temperature in the modified Tyrode’s solution used above. Cells were then placed in an experimental chamber (volume = 0.5 ml) filled with Tyrode’s solution and mounted on a Leica 6000 DMI inverted microscope attached to a Leica SP5 resonant scanning laser confocal system (Leica, GERMANY). To obtain linescan (x-t) images of rhod-2 fluorescence, we used a 63x/1.2 CS water objective (Leica s/n: 15506279) and the x-t mode of the SP5 scanner (2 ms/line) during excitation using an Argon/Krypton laser (ex: 488 nm; em: >510 nm). Fluorescence data were analyzed using LAS AF software (v 2.4, Leica, GERMANY).

The confocal system was attached electronically to a patch clamp system for synchronized stimulation of cells and recording of action potentials during imaging. Cells were patched with gently fire-polished glass microelectrodes (World Precision Instruments, Inc. WPI TWF 150) filled with internal solution containing (in mM): 130 KCl, 10 NaCl, 10 HEPES, 0.05 cAMP, 5 phosphocreatine, 5 Mg-ATP, (pH 7.2). We used the bridge mode of an Axopatch 900A amplifier (Molecular Devices) to stimulate (0.2 Hz) and record action potentials using a Digidata 1440 analogue to digital converter (Molecular Devices) under the control of the Clampex component of the pClamp 10.2 system (Molecular Devices). Data were analyzed using the Clampfit component of the pClamp 10.2 system.
