# Supplementary material for: Characterization and Therapeutic Potential of Induced Pluripotent Stem Cell-Derived Cardiovascular Progenitor Cells
Source: PLoS One. 2012 Oct 9;7(10):e45603. doi: 10.1371/journal.pone.0045603 (PMC3467279; doi:10.1371/journal.pone.0045603)
Supplement: Table S1 — Primary and secondary antibodies. (DOC) [file pone.0045603.s006.doc]

| **Table S1:** Primary and Secondary Antibodies. | | |
| --- | --- | --- |
| **(I) Primary Antibodies** | | |
| **Antibody** | **Catalogue number and dilution** | **Source** |
| alpha smooth muscle actin (αSMA) | ab5694; 1:400 | abcam, Cambridge, MA, [www.abcam.com](http://www.abcam.com/) |
| Flt1 | ab2350; IF 1:1000, FACS 1:50 | abcam |
| Flt4 | ab27278; IF 1:1000, FACS 1:50 | abcam |
| Troponin C | sc-48347; 1:50 | Santa Cruz Biotechnology, Inc., Santa Cruz, CA, www.scbt.com |
| ckit | A4502; 1:1000 | Dako USA, Carpinteria, [www.dakousa.com](http://www.dakousa.com/) |
| c-myc | sc-788; 1:500 | Santa Cruz Biotechnology |
| Nkx2.5 | ab22611; 1:250 | abcam |
| Flk1 | sc-6251; 1:1500 | Santa Cruz |
| Isl1 | 40-206; IF and FACS, 1:250 | Developmental Studies Hybridoma Bank (DSHB), Iowa City, IA, http://dshb.biology.uiowa.edu |
| GFP | sc-101525; 1:200 | Santa Cruz Biotechnology |
| Pecam1 (CD31) | 550274; 1:50 | BD Pharmingen, San Diego, CA, www.bdbiosciences.com/pharmingen |
| Oct4 | sc-8629; 1:500 | Santa Cruz Biotechnology |
| Map2 | ab11267; 1:400 | abcam |
| Smi32 (NEFH) | Smi-32R-500; 1:400 | Covance Inc.  Princeton, NJ, www.covance,com |
| **(II) Secondary Antibodies** | | |
| Antibody | **Conjugation and dilution** | **Source** |
| Alexa Fluor 594-conjugated goat-anti mouse IgG (H+L) | goat-anti rabbit IgG (H+L); 1:250 | Molecular Probes, Eugene, OR, probes.invitrogen.com |
| Alexa Fluor 647-conjugated goat-anti mouse IgG (H+L) | goat-anti rat IgG (H+L); 1:250 | Molecular Probes |
| Alexa Fluor 488-conjugated goat-anti mouse IgG (H+L) | goat-anti mouse IgM (H+L); 1:250 | Molecular Probes |

1. Schenke-Layland K, Rhodes KE, Angelis E, Butylkova Y, Heydarkhan-Hagvall S, et al. (2008) Reprogrammed mouse fibroblasts differentiate into cells of the cardiovascular and hematopoietic lineages. *Stem Cells.* 6(6): 1537-1546.
